# Radioligand therapy using [^177^Lu]Lu-PSMA-617 in mCRPC: a pre-VISION single-center analysis

**DOI:** 10.1007/s00259-020-04703-3

**Published:** 2020-02-16

**Authors:** Robert Seifert, Katharina Kessel, Katrin Schlack, Matthias Weckesser, Martin Bögemann, Kambiz Rahbar

**Affiliations:** 1 Department of Nuclear Medicine, University Hospital Münster, Albert-Schweitzer-Campus 1, D-48149 Münster, Germany; 2 Department of Urology, University Hospital Münster, Münster, Germany

**Keywords:** ^177^Lu-PSMA-617, mCRPC, Radioligand therapy, VISION Trial

## Abstract

**Background:**

Radioligand therapy with [^177^Lu]Lu-PSMA-617 is efficacious for the treatment of patients with metastasized castration-resistant prostate cancer (mCRPC). Various studies have evaluated the efficacy and safety of [^177^Lu]Lu-PSMA-617 using a dose of 6.0 GBq and an 8-week therapy interval. However, the first prospective phase III trial (VISION) plans to use an elevated cumulative dose by applying 7.5 GBq in a 6-week interval. The aim of the present study was to compare safety and efficacy of the two aforementioned [^177^Lu]Lu-PSMA-617 therapy regimes (7.5 GBq every 6 weeks vs. 6.0 GBq every 8 weeks).

**Methods:**

A total number of 78 consecutive patients with mCRPC and a history of first-line chemotherapy were included in this retrospective analysis. The outcome of patients treated with 6.0 GBq [^177^Lu]Lu-PSMA-617 per cycle (*n* = 37) were compared with those treated with 7.5 GBq (*n* = 41) per cycle. The median therapy intervals were 8.4 weeks (6.0 GBq group) vs. 6.5 (7.5 GBq group). PSA response, PSA progression-free survival (PSA-PFS), overall survival, and adverse events were evaluated and compared between both groups. Chi-squared test, Kaplan Meier estimates, Cox regression, and log-rank test were used. The highest decline from pretherapeutic PSA levels was measured as percentage (best PSA response) and compared between groups by Wilcoxon test.

**Results:**

There was no significant difference comparing the rate of > 50% PSA decline or best PSA response between the 6.0 GBq and 7.5 GBq group (35% vs. 54%, *p* = 0.065; and − 40.2% vs. − 57.8%, *p* = 0.329). The median estimated survival and PSA-PFS did not significantly differ between the 6.0 GBq and 7.5 GBq groups as well (11.3 vs. 12.7 months, *p* = 0.384; and 9.5 vs. 12.3 months, *p* = 0.258). There was no significant difference regarding the change of kidney, liver, and blood cell parameters under therapy between the treatment groups.

**Conclusion:**

Higher cumulated doses of [^177^Lu]Lu-PSMA-617 were well tolerated and caused no significantly increased rate of adverse reactions. Moreover, 7.5 GBq of [^177^Lu]Lu-PSMA-617 every 6 weeks causes slightly higher, though not statistically significant, response rates and seems therefore to be the preferable treatment regime. However, future studies are needed to elucidate the dose-related efficacy of [^177^Lu]Lu-PSMA-617 as a way to personalized medicine.

## Introduction

Radioligand therapy regimes targeting the prostate-specific membrane antigen (PSMA) by [^177^Lu]Lu-PSMA-617 have become a promising option for patients with metastasized castration-resistant prostate cancer (mCRPC) and have been extensively evaluated by multiple retrospective studies [1–6]. A prospective phase II trial at the Peter McCullum Center, Australia, was giving additional evidence to the efficacy and favorable toxicity profile of [^177^Lu]Lu-PSMA-617 [7]. Furthermore, the prospective phase III registrational trial (VISION) is currently running to evaluate the efficacy and safety in these patients and aims to bring this promising therapeutic to approval [8].

Initial studies like the German Multicenter Study predominantly employed a target dose of 6.0 GBq (range 2–8 GBq) of lutetium every 8 weeks to evaluate the safety and efficacy of ^177^Lu-PSMA-617 [1]. Yet, in recent studies, the actual applied dose per cycle varies from 6.0 to 7.5 GBq, and some groups have shortened the therapy interval to 6 weeks [7]. The dose escalation toward 7.5 GBq was pursued analogously to the NETTER trial, which recommended the use of 200 mCi (~ 7.5 GBq) for the treatment of neuroendocrine cancer [9]. Yet, to date, no systematic evaluation of the efficacy of 6.0 GBq every 8 weeks vs. 7.5 GBq every 6 weeks has been reported. Therefore, it is hard to assess the dose-specific efficacy of [^177^Lu]Lu-PSMA-617.

The study protocol of the VISION trial requests the administration of 7.5 GBq every 6 weeks. As outlined above, it is currently unclear which efficacy and safety profile is to expect from the VISION trial protocol: Will it result in a higher rate of adverse reactions or in an improved efficacy compared with initial studies, which used 6.0 GBq every 8 weeks? Moreover, the VISION trial requires previous treatment by at least one taxane chemotherapeutic, which is known to influence the response to [^177^Lu]Lu-PSMA-617 therapy [10]. The combination of mixed target doses per cycle (6.0 or 7.5GBq), mixed therapy interval (6 or 8 weeks), and mixed patient collectives (naïve to or pre-treated with taxanes) in current literature is challenging for the clinical decision making.

We hypothesize that higher cumulated doses (7.5 GBq [^177^Lu]Lu-PSMA-617 every 6 weeks) have no systematic effect on response or survival compared with 6.0 GBq every 8 weeks and that the efficacy of newer therapy regimens should therefore be comparable with the initial [^177^Lu]Lu-PSMA-617 studies. Therefore, the aim of the present study was to compare two patient collectives that were both previously treated with taxane chemotherapy and received 6.0 GBq (8-week interval) or 7.5 GBq (6-week interval) of [^177^Lu]Lu-PSMA-617. To our best knowledge, this study is thereby applying an adapted version of the VISION trial protocol for the first time in a large patient collective, which is of importance to estimate the anticipated efficacy (1) and to investigate, if the efficacy is dose dependent and safe (2).

## Methods

### Inclusion criteria and patient stratification

Patients with prostate cancer were referred to [^177^Lu]Lu-PSMA-617 therapy after critical evaluation in an interdisciplinary tumor board on a case by case bases. Patients were eligible for therapy, if the established inclusion criteria were met: presence of mCRPC; hemoglobin > 8 g/dL; leukocytes > 2.0 × 10^9^/L; platelets > 75,000/μL; creatinine < 2.0 mg/dL; aspartate transaminase (AST) and alanine transaminase (ALT) < 5 times of upper limit of normal, history of at least one line of chemotherapy (taxane) as well as history of at least one line of new generation anti androgenic therapy (abiraterone or enzalutamide, if tolerated) [11].

Patients referred to our department for [^177^Lu]Lu-PSMA-617 from December 2014 to December 2018 were considered for this study. Patients were treated with either 6.0 GBq every 8 weeks or 7.5 GBq every 6 weeks. The choice of treatment regime was independent from performance status or tumor volume and solely attributed to changed therapy protocols at our department caused by the announcement of the VISION trial protocol.

The following inclusion criteria were employed to clearly distinguish two groups with regard to given therapeutic activity. These inclusion criteria were needed to minimize the influence of varying radiochemical yield:

1. Mean activity per patient (of all cycles): ≥ 6.9 GBq (7.5 group) or < 6.9 GBq (6.0 group) (patients receiving 7.5 GBq were treated with a 6-week interval)
2. Range (=difference between smallest and largest doses) of all cycles of a patient: < 1.0 GBq
3. Maximum interval between cycles: 6 months (patients with a therapeutic pause between the administration of two [^177^Lu]Lu-PSMA-617 cycles were not included)

A total number of 78 (37 patients, 6.0 GBq; 41 patients, 7.5 GBq group) patients met these criteria and were thus included in this retrospective analysis; a detailed patient characterization is given in Table 1.

**Table 1:** Patient characteristics

Characteristics	7.5 GBq group (*n* = 41)		6 GBq group (*n* = 37)	
	Median (IQR)	% (*n*)	Median (IQR)	% (*n*)
Age (years)	68.7 (10.4)	–	72.7 (10.0)	–
Gleason score	8 (2)	–	9 (2)	–
PSA (ng/mL)	195.0 (479.3)	–	420.6 (1061.1)	–
ECOG				
0	–	32.4% (12)	–	25.7% (9)
1	–	56.8% (21)	–	54.3% (19)
2	–	10.8% (4)	–	17.1% (6)
3	–	0% (0)	–	2.9% (1)
Alkaline phosphatase (U/L)	129.5 (171.0)	–	185.0 (218.5)	–
Lactase dehydrogenase (U/L)	338.5 (210.5)	–	331.0 (228.0)	–
Hemoglobin (g/dL)	11.4 (1.9)	–	10.1 (2.5)	–
Site of metastases				
Bone	–	87.5% (35)	–	97.3% (36)
Lymph nodes	–	77.5% (31)	–	83.8% (31)
Liver	–	17.5% (7)	–	30.6 (11)
Lung	–	10.0% (4)	–	28.6 (10)
Brain	–	5.0% (2)	–	8.3% (3)
Previous therapy of mCRPC				
Docetaxel	–	100% (41)	–	100% (37)
Cabazitaxel	–	41.5% (17)	–	16.2% (6)
Abiaterone or enzalutamide	–	97.6% (40)	–	94.6% (35)
Abiaterone and enzalutamide	–	70.7% (29)	–	73.0% (27)
Abiaterone	–	90.2% (37)	–	78.7% (29)
Enzalutamide	–	78.0% (32)	–	89.2% (33)
Median number of cycles	4 (4)	–	3 (2)	-
Median Lu-PSMA activity per cycle (GBq)	7.4 (0.2)	–	6.1 (0.1)	–
Median Lu-PSMA activity range of all cycles (GBq)	0.3 (0.4)	–	0.4 (0.3)	
Median cumulated Lu-PSMA activity of all cycles (GBq)	29.6 (28.8)		18.2 (12.6)	
Median therapy interval (weeks)	6.5 (1.0)	–	8.4 (2.4)	–
Time from prostate cancer diagnosis to start of PSMA therapy (years)	7.2 (7.4)	–	6.9 (8.2)	–

*IQR* interquartile range

### [^177^Lu]Lu-PSMA-617 therapy preparation and administration

The conjugation of lutetium (ITG Isotopes Technology, Garching, Germany) and PSMA-617 (ABX advanced biochemical compounds, Radeberg, Germany) was described previously [12]. Therapy was administered in a 6- to 8-week interval until tumor progression, severe adverse reactions, death, or an altered therapy strategy. A prophylactic cooling of the salivary glands was conducted with growing expertise in administration, as described elsewhere [3].

### Therapy monitoring

Blood samples and their toxicity were evaluated at least at each cycle administration. Adverse reactions were evaluated according to the Common Terminology Criteria for Adverse Events (CTCAE) Version 5.0. The following upper/lower limits of normal were used for the grading according to CTCAE: leukocytes < 3.9 × 10^9^/L; hemoglobin < 13.5 g/dL; platelets < 166,000 /μL; creatinine 1.4 mg/dL; AST 50 U/L; ALT 50 U/L.

PSA progression-free survival (PFS) was defined by the Prostate Cancer Clinical Trials Working Group 3 (PCWG3) as a PSA incline of 25% from baseline (minimal 2 ng/mL absolute incline), which has to be confirmed by a second measurement (3 weeks later) [13]. Biochemical response was defined in accordance with PCWG3 as 50% PSA decline from baseline [13]. Additionally, the rate of 30% PSA decline from baseline was reported in accordance with previous publications [14].

### Statistics and data analysis

MATLAB R2018b (The MathWorks, MA, USA), Excel (Microsoft, WA, USA), and SPSS Statistics 24 (IBM, NY, USA) were used for descriptive statistics and testing. The non-paired, non-parametric Mann-Whitney *U* test or the Chi-squared test was employed to compare the therapy groups. Overall survival and PSA-PSF were investigated using Kaplan Meier and Cox regression estimates; log-rank test was used to test for statistical significance of estimated survival. *p* values < 0.05 were regarded as statistically noticeable.

## Results

### Patient characteristics

Depending on the employed therapy scheme, two patient groups were formed: the 6.0 GBq group with a median applied activity of 6.1 GBq and the 7.5 GBq group with a median activity of 7.4 GBq per cycle. The median range of administered activity over all cycles was 0.4 GBq for the 6.0 GBq group or 0.3 GBq for the 7.5 GBq group, respectively. The cycle interval was shortened concomitantly with the increased therapy activity: the 6.0 GBq group was treated with a median interval of 8.4 weeks, whereas the 7.5 GBq group had a median interval of 6.5 weeks.

All patients were previously treated with docetaxel. Both abiraterone and enzalutamide were subsequentially applied in 73% of patients (6.0 GBq group) or 71% of patients (7.5 GBq group). The 6.0 GBq group had a notably higher rate of liver (31 vs. 18%, *p* = 0.280) and lung (29 vs. 10%, *p* = 0.072) metastases. The median time from prostate cancer diagnosis to start of PSMA therapy was comparable (6.9 years for the 6.0 GBq group vs. 7.2 years, *p* = 0.685). Please see Table 1 for details.

### PSA response

There was no significant difference between the 6.0 GBq and 7.5 GBq groups regarding the 50% PSA decline (35 vs. 54%, *p* = 0.065), 30% PSA decline (70 vs. 68%, *p* = 1.000), or the PSA-PFS (38 vs. 49 weeks, *p* = 0.258). Details are given in Table 2.

**Table 2:** PSA response evaluation

	7.5 GBq group (*n* = 41)		6 GBq group (*n* = 37)		
	Median (IQR)	% (*n*)	Median (IQR)	% (*n*)	*p*
Best PSA response (> 50% decline)	–	53.7% (22)	–	35.1% (13)	0.065
Best PSA response (> 30% decline)	–	68.3% (28)	–	70.3% (26)	1.000
Best PSA response	− 57.8 (74.5) %	–	− 40.2 (44.6) %	–	0.329
Initial PSA decline (min 6–9 weeks after first therapy)	− 37.0 (73.5) %	–	− 31.6 (45.4) %	–	0.414
PSA progression-free survival (weeks)	49.2 (40.7–57.7)*	–	38.0 (29.4–46.5) *	–	0.258

*Mean and 95% confidence interval; *IQR* interquartile range

### Overall survival

**Fig. 1:**
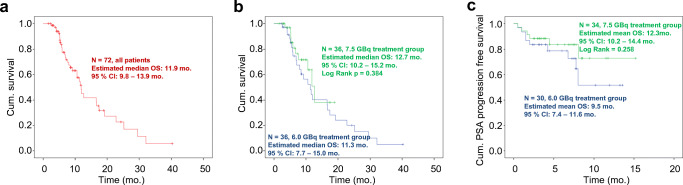
Survival and progression-free survival. Panel **a** depicts the survival of all included patients. Additionally, the group-specific survival (**b**) and PSA progression-free survival (PSA-PFS, **c**) are shown

For all patients, the estimated overall survival was 47 weeks. The estimated overall survival did not differ between the 6.0 GBq and 7.5 GBq groups (45 vs. 51 weeks, *p* = 0.384 | HR 1.404, 95%CI 0.650–3.031, reference category = 6 GBq group; *p* = 0.388). Please see Fig. 1 for details.

### Safety evaluation

Adverse reactions are presented in Tables 3 and 4. The changes in white blood cell, hemoglobin, platelet, and aspartate aminotransferase and alanine aminotransferase levels during therapy were not significantly different between the activity groups. Notably, 32% stated grade 1 or 2 xerostomia in the 6.0 GBq group, whereas no xerostomia was reported in the 7.5 GBq group. Consequent cooling of salivary glands was only introduced in our department after initial administrations of [^177^Lu]Lu-PSMA-617, which were all covered by the 6.0 GBq group. Apart from xerostomia and eye dryness, a noticeable fraction of patients suffered from grade 3 or 4 anemia.

**Table 3:** Adverse reactions according to CTCAE v5.0

	7.5 GBq group (*n* = 41)		6 GBq group (*n* = 37)	
	Occurrence of grade 1 + 2 (%)	Occurrence of grade 3 + 4 (%)	Occurrence of grade 1 + 2 (%)	Occurrence of grade 3 + 4 (%)
Leukopenia	48.8	2.4	48.6	8.1
Anemia	26.8	24.4	35.1	21.6
Thrombocytopenia	34.1	2.4	40.5	8.1
Creatinine elevation	7.5	0.0	13.5	0.0
Aspartate aminotransferase elevation	20.0	7.5	37.8	0.0
Alanine aminotransferase elevation	10.0	0.0	13.5	0.0
Eye dryness	9.8	0.0	10.8	0.0
Xerostomia	0.0	0.0	32.4	0.0
Nausea	9.8	0.0	10.8	0.0
Diarrhea	17.1	0.0	18.9	0.0

*IQR* interquartile range

**Table 4:** Adverse reactions—change of blood parameters under ^177^Lu-PSMA-617 therapy

	7.5 GBq group (all cycles)	6 GBq group (all cycles)	
	Median (IQR)	Median (IQR)	*p*
WBC	− 35.9 (21.1) %	− 38.5 (18.0) %	0.555
HB	− 10.6 (12.7) %	− 11.1 (17.6) %	0.466
Platelets	− 34.5 (26.6) %	− 33.9 (18.9) %	0.635
Creatinine	+ 16.7 (30.9) %	+ 16.2 (30.3) %	0.815
Aspartate aminotransferase	+ 18.2 (76.0) %	+ 38.9 (81.1) %	0.153
Alanine aminotransferase	+ 16.7 (80) %	+ 38.2 (79.3) %	0.230

*WBC*, white blood cell levels; *HB*, hemoglobin levels; *IQR*, interquartile range

## Discussion

The aim of the present study was the comparison of two different doses of [^177^Lu]Lu-PSMA-617 regarding safety and efficacy for the therapy of end-stage mCRPC. Outcome and adverse reactions of patients receiving 6.0 GBq of [^177^Lu]Lu-PSMA-617 every 8 weeks are retrospectively compared with those of patients receiving 7.5 GBq [^177^Lu]Lu-PSMA-617 every 6 weeks. This comparison is clinically needed, as initial studies that evaluated ^177^Lu-PSMA-617 employed 6.0 GBq (8-week interval), whereas the currently enrolling prospective VISION trial protocol is demanding the usage of 7.5 Gbq (6-week interval) [1, 8]. To date, safety and efficacy of [^177^Lu]Lu-PSMA-617 given as 7.5 GBq doses every 6 weeks has not been studied in large cohorts. In contrast, the Münster group adopted the VISION trial protocol in the past and could thus show here that higher cumulated doses of [^177^Lu]Lu-PSMA-617 do not cause significant changes in the adverse reaction profile. Moreover, higher cumulated doses of [^177^Lu]Lu-PSMA-617 caused a noticeable improved PSA response, PSA-PFS, and overall survival of mCRPC patients. However, efficacy and overall survival did not significantly differ between both treatment regimes.

The VISION trial is the first prospective phase III trial investigating [^177^Lu]Lu-PSMA-617 [8]. Patients suffering from mCRPC that were pretreated with a taxane and abiraterone or enzalutamide are eligible. For all enrolled patients, the VISION trial requests the administration of the hitherto not given next-generation antiandrogen substance (i.e., abiraterone or enzalutamide). Thereafter, patients are randomized to either receive 7.5 GBq [^177^Lu]Lu-PSMA-617 every 6 weeks up to 6 cycles or not.

The determination of the optimal amount of activity for [^177^Lu]Lu-PSMA-617 is not an easy task. Initial studies investigating the usage of [^177^Lu]Lu-PSMA-617 were highly influenced by the dosimetry data of ^131^I-MIP-1095, which employed a dose of 4.8 GBq [15]. Consequently, the first retrospective study evaluating the efficiency and toxicity of [^177^Lu]Lu-PSMA-617 administered 4.0 GBq [16]. However, the administered doses were escalated after the first dosimetry studies were conducted [16, 17]. One of the first of these studies was done by Delker et al., who suggested the usage of 6.0 GBq for therapy [17]. This was corroborated later by Fendler et al. in a larger patient cohort [18]. The dose escalation toward 6.0 GBq seemed possible due to the different isotope characteristics of [^177^Lu]lutetium compared with [^131^I]iodine and became the reference standard in most departments.

It was shown that 6.0 GBq ^177^Lu-PSMA-617 exhibits greater tumor to kidney ratios than 7.5 GBq [^177^Lu]Lu-DOTATATE, which was extensively evaluated in the NETTER trial [9, 18]. Motivated by the promising dosimetry results, further dose escalations of [^177^Lu]Lu-PSMA-617 seemed reasonable. Therefore, Rathke et al. analyzed the effect of escalated doses of ^177^Lu-PSMA-617 (4.0, 6.0, 7.4, 9.3 GBq) on efficacy and toxicity. However, the authors predominantly discussed the benefit of dose escalation up to 9.4 GBq in the context of bone marrow toxicity and only included ten patients per dose group. Thus, a formal dose-escalating study to define a dose with an optimal efficacy-toxicity profile has not been done to this day. Additionally, transferability of the results of Rathke et al. to the clinical routine is impaired, as patients with end-stage mCRPC are rather evaluated for [^225^Ac]Ac-PSMA ligands and therefore not included in the analysis [19]. Moreover, only 40–50% of enrolled patients had received taxane chemotherapy, which is violating the VISION trial enrollment criteria. Finally, an 8-week therapy interval was used, which is in contrast to the VISION trial protocol; therefore, the study does not allow making conclusions of the anticipated efficacy or toxicity of higher cumulated doses. Interestingly, the authors reported PSA response (> 50% decline) rates of 30% and 50% for 6.0 and 7.5 GBq, respectively, which is in line with the results presented here.

Hofman et al. investigated the usage of [^177^Lu]Lu-PSMA-617 in a prospective study employing a mean activity of 7.5 GBq every 6 weeks [7]. However, the study protocol was designed for only 4 cycles, allowed the rechallenge of patients, and applied a dose that was dependent on the tumor burden and body weight. Therefore, direct transferability to a fixed 7.5 GBq scheme is not warranted. The preselection criteria (30% excluded) additionally impaired comparability with the VISION trial protocol. Finally, not all enrolled patients had received first-line taxane chemotherapy.

The treatment with [^177^Lu]Lu-PSMA-617 seems to be less effective if chemotherapy, especially second-line chemotherapy, was applied previously [2, 10]. However, it still has to be determined if this is caused by a meta-phenomenon or if the chemotherapy itself selects more aggressive tumor cells. Barber et al. has shown that first-line taxane chemotherapy is significantly decreasing the overall survival of mCRPC patients treated with [^177^Lu]Lu-PSMA-617 [10]. Kessel et al. could demonstrate that second-line chemotherapy is a negative predictor in a highly pretreated patient collective [2]. Therefore, it seems necessary to only include patients pretreated with at least a first chemotherapy to estimate the efficacy of the VISION trial protocol.

Interestingly, the results of the present study indicate that higher cumulated doses (7.5 GBq, 6-week interval) did not lead to a significantly improved efficacy. On the contrary, the reported PSA response rates are similar to those of previously reported studies and only slightly better compared with the 6.0 GBq group. However, this is in line with a previous dose-escalating study by Rathke et al. (up to 9.3 GBq) which could not demonstrate a significant increase of response compared with 6.0 GBq [20]. This might partly be explained by immunological effects of radioligand therapies, which do not follow a linear dose-efficacy relationship. It might be possible that radioligand therapies cause an inflammatory reaction in the tumor (I) and lead to an antigen release (II), which taken together increases the T cell receptor variability and boost an immunogenic anti-tumor effect [21]. Future studies focusing on tumor inflammation caused by radioligand therapies seem warranted.

Response rates of the 7.5 GBq [^177^Lu]Lu-PSMA-617 regime were noticeably higher compared with the 6.0 GBq regime, while the adverse reactions profile remained modest. Therefore, it seems reasonable to prefer the 7.5 GBq regime in most patients. However, especially in patients with limited hematopoietic reserve and extensive bone metastases, it might be advisable to reduce the employed therapeutic dose to 6.0 GBq.

A considerable fraction of patients suffered from grade 3 and 4 anemia. However, it must be noted that the hematologic reserve was limited in these end stage, heavily pretreated patient collective (> 87% suffered from bone metastases, median baseline hemoglobin was lowered to 11.4 and 10.1 g/dL for the 7.5 and 6.0 GBq group, respectively). Therefore, severe anemia seems to be rather attributed to the end-stage cancer rather than to a therapeutic side effect.

This study faces some limitations. It was designed as a retrospective analysis thereby facing biases of non-randomization. The patient collective referred to [^177^Lu]Lu-PSMA-617 therapy might have changed over time due to a broad clinical acceptation of the therapy caused by its efficacy and tolerability. This bias might have been aggravated, as patients treated with 6.0 GBq (8-week interval) were referred to therapy in the initial days of [^177^Lu]Lu-PSMA-617 therapy, whereas patients treated with 7.5 GBq (6-week interval) are enrolled more recently. Therefore, prospective studies addressing this question are mandatory to optimize the application of the amount of activity and the respective frequency.

## Conclusion

The toxicity profile of higher cumulated [^177^Lu]Lu-PSMA-617 doses utilizing 7.5 GBq (6-week interval) is not significantly different compared with 6.0 GBq (8-week interval) in a taxane and abiraterone/enzalutamide pretreated mCRPC patient collective. The efficacy of [^177^Lu]Lu-PSMA-617 therapy using higher cumulated activity is noticeably higher, although not statistically significant. Therefore, future studies evaluating the dose-specific efficacy of [^177^Lu]Lu-PSMA-617 seem warranted.
